# Is it possible to improve residents breaking bad news skills? A randomised study assessing the efficacy of a communication skills training program

**DOI:** 10.1038/sj.bjc.6605749

**Published:** 2010-07-13

**Authors:** A Liénard, I Merckaert, Y Libert, I Bragard, N Delvaux, A-M Etienne, S Marchal, J Meunier, C Reynaert, J-L Slachmuylder, D Razavi

**Affiliations:** 1 Clinique de Psycho-Oncologie et des Soins Supportifs, Institut Jules Bordet, Brussels, Belgium; 2 Unité de Recherche en Psychosomatique et Psycho-Oncologie, Faculté des Sciences Psychologiques et de l'Éducation, Université Libre de Bruxelles, Avenue F Roosevelt, 50 – CP 191, B-1050 Brussels, Belgium; 3 Secteur ≪Psychologie de la santé≫, Faculté de Psychologie, Université de Liège, Liège, Belgium; 4 Service de Psychologie, Hôpital Erasme, Brussels, Belgium; 5 CAM (Training and Research Group), Brussels, Belgium; 6 Service de Psychosomatique, Faculté de Psychologie et des Sciences de l'Éducation, Université Catholique de Louvain, Louvain-la-Neuve, Belgium

**Keywords:** breaking bad news, communication skills, training, residents, randomised study

## Abstract

**Background:**

This study aims to assess the efficacy of a 40-h training programme designed to teach residents the communication skills needed to break the bad news.

**Methods:**

Residents were randomly assigned to the training programme or to a waiting list. A simulated patient breaking bad news (BBN) consultation was audiotaped at baseline and after training in the training group and 8 months after baseline in the waiting-list group. Transcripts were analysed by tagging the used communication skills with a content analysis software (LaComm) and by tagging the phases of bad news delivery: pre-delivery, delivery and post-delivery. Training effects were tested with generalised estimating equation (GEE) and multivariate analysis of variance (MANOVA).

**Results:**

The trained residents (*n*=50) used effective communication skills more often than the untrained residents (*n*=48): more open questions (relative rate (RR)=5.79; *P*<0.001), open directive questions (RR=1.71; *P*=0.003) and empathy (RR=4.50; *P*=0.017) and less information transmission (RR=0.72; *P*=0.001). The pre-delivery phase was longer for the trained (1 min 53 s at baseline and 3 min 55 s after training) compared with the untrained residents (2 min 7 s at baseline and 1 min 46 s at second assessment time; *P*<0.001).

**Conclusion:**

This study shows the efficacy of training programme designed to improve residents' BBN skills. The way residents break bad news may thus be improved.

Breaking bad news (BBN) to patients is one of the physicians' stressful clinical tasks. This task is frequent for physicians caring for cancer patients. Breaking bad news has an impact not only on patients (Fallowfield and Jenkins, 2004; Schmid Mast *et al*, 2005; Lienard *et al*, 2006) but also on physicians' emotional state (Ptacek *et al*, 1999; Fallowfield and Jenkins, 2004). To make BBN as bearable as possible for patients, physicians have to use effective communication skills and to be able to manage their stress linked with this task. This may be difficult for residents. Residents often feel that they do not use adequate communication skills.

Guidelines and recommendations about how physicians should lead BBN consultations have been published (Girgis and Sanson-Fisher, 1995; Baile *et al*, 2000; Fallowfield and Jenkins, 2004). On the basis of these guidelines, BBN has been described as a three-phase process including different tasks. The first phase is devoted to preparing the patient for bad news delivery (‘pre-delivery phase’) by assessing what the patient knows, understands and feels about the situation. The second phase is devoted to delivering the bad news (delivery phase). Bad news delivery should be done precisely and concisely. The third phase is devoted to giving informational and emotional support to the patient (‘post-delivery phase’). Each of these three phases is a complex task requiring the use of numerous communication skills for which physicians have not been trained enough. Although as Eggly *et al* (2006) have argued that these three phases only infrequently occur in actual cancer clinical interactions, this three-phase structure has several advantages. First, it is a useful structure for training junior physicians. Second, it is a structure that allows physicians in every interaction to determine whether they support or inform patients prematurely in the context of their individual needs. An appropriate preparation during the pre-delivery phase allows personalising emotional and informational support during the post-delivery phase. Third, this structure is highly relevant to BBN, the specific situation that is still one of the most stressful and challenging communication tasks for physicians.

Communication skills training programmes using learner-centred, skills-focused and practise-oriented techniques have been found to be effective for physicians (Fallowfield *et al*, 2002; Razavi *et al*, 2003; Roter 2003). Some communication skills training programmes have been organised for residents (Robbins *et al*, 1979; Langewitz *et al*, 1998; Smith *et al*, 1998; Oh *et al*, 2001; Yedidia *et al*, 2003; Losh *et al*, 2005; Alexander *et al*, 2006; Back *et al*, 2007). Among them, the study by Back *et al* (2007) found that after a communication skills workshop, residents acquired bad news skills, specifically assessment and empathy skills. Only three of these programmes, however, were assessed for their efficacy in randomised controlled studies (Robbins *et al*, 1979; Langewitz *et al*, 1998; Smith *et al*, 1998): these studies have shown improvements regarding the observed communication skills during consultations with simulated patients (Langewitz *et al*, 1998; Smith *et al*, 1998) and with actual patients (Robbins *et al*, 1979; Smith *et al*, 1998). Finally, two controlled, but non-randomised studies, have shown an improvement in residents' use of communication skills (Oh *et al*, 2001; Alexander *et al*, 2006). Only one of these studies has focused specifically on the learning of BBN (Alexander *et al*, 2006).

Given the limited experience in this area of training, there is still a need to study the efficacy of training programmes focusing on BBN and specifically designed for residents. The aim of this study was to assess the efficacy of such a programme in a randomised controlled design during a simulated patient BBN consultation. A specific training programme has been designed for residents specialising in various disciplines (Belgian Interuniversity Curriculum – communication skills training (BIC-CST)) (Bragard *et al*, 2006). One of the main aims of the training programme was to promote knowledge and the use of communication skills needed to break the bad news. It (BIC-CST) is a 40-h training programme, which is learner centered, skill focused, practice oriented and tailored to participants' needs.

Belgian Interuniversity Curriculum – communication skills training should have an impact on residents' BBN skills during a simulated patient consultation not only on communication contents but also on the BBN process. First, it was hypothesised that BIC-CST would lead to an increase in residents' use of assessment and supportive skills and a decrease in the information transmitted. This would allow residents to be more patient centred by avoiding to overload patients with information that they are not able to process (Merckaert *et al*, 2009). Second, it was hypothesised that BIC-CST would lead the simulated patients to express their concerns more often. Third, it was hypothesised that BIC-CST would change the three-phase BBN process. However, BIC-CST would have an impact on the time allocated to each of the three phases of the BBN process (the pre-delivery, delivery and post-delivery phases) and on the other hand, BIC-CST would have an impact on the way the residents deliver bad news. More precisely, it was hypothesised that residents do not assess what patients feel, know and understand about their situation sufficiently (a too short pre-delivery phase) and that residents deliver bad news not concisely (a too long delivery phase) and precisely enough. After training, an increase in the duration of the pre-delivery phase, a decrease in the duration of the delivery phase and an improvement in the way the bad news is delivered should thus be found.

## Materials and methods

### Subjects

To be included in this study, residents had to speak French, show an interest for communication skill training and to be willing to participate in the training programme and its assessment procedure. Physicians also had to have worked, to work or to have the project of working with cancer patients (part or full time). Residents already participating in another psychological training programme during the assessment and training periods were excluded from the study.

### Study design and assessment procedure

The efficacy of the BIC-CST was assessed in a study allocating residents after the first assessment time to a 40-h training programme (training group) or to a waiting list (waiting-list group), according to a computer-generated randomisation list. Assessments were scheduled before the randomisation and after the training programme for the training group and 8 months after baseline for the waiting-list group. At each assessment time, the procedure included, among others, a BBN consultation with a simulated patient. The local ethics committee approved the study.

### Training programme

Belgian Interuniversity Curriculum – communication skills training included 30-h of communication skills and 10-h of stress management training (Bragard *et al*, 2006). Sessions were spread over an 8-month period and were organised bimonthly in small groups (up to seven participants). The communication skill training module consisted of a 17-h communication skill training focusing on two-person consultations including six sessions, a 10-h communication skills training focusing on three-person consultations (i.e. where a relative accompanies the patient) including three sessions and a last 3-h session promoting integration of learned communication and stress management skills. Among these 30 h, a 1-h session focused on theoretical information. In the other sessions, residents were invited to practice communication skills through predefined role plays and through role plays based on the clinical problems brought up and played by the participants. Residents were given immediate feedback on the communication skills performed during the role plays. Themes of predefined role plays were BBN (breaking cancer diagnosis and discussing transition from cure to palliation). During the course, the facilitator introduced gradually the three phases of the BBN process. The choice of the skills taught was based on the results of studies showing the positive impact of using specific communication skills on patients' disclosure of concerns (Maguire *et al*, 1996). The training programme was designed on the basic premises that, to adapt information and support giving to individual patients' concerns and needs, physicians need to assess those concerns and needs first. The rationale underlying the training programme included the need to handle BBN consultations step by step. The first step, corresponding to the pre-delivery phase, should therefore be devoted to assessing what the patient knows, understands and feels about the situation. The second step, corresponding to the delivery phase, should be devoted to precise and clear information transmission. The third step, corresponding to the post-delivery phase, should include an assessment of patients' emotional status and understanding. Depending on this assessment, residents should consider to support patients if they are distressed (emotional support) or to further inform patients if they request more information (informational support) (Razavi and Delvaux, 2008). It should be noted that physicians were not specifically taught to increase the pre-delivery phase and decrease the post-delivery phase. These changes in the time allocated to the three phases were expected to result from the changes in the BBN process.

### Simulated patient consultation

Residents' communication skills were assessed in a simulated patient consultation. Simulated patient consultations have been described as a valid method to study the communication style (Roter *et al*, 1995). Consultations were audiotaped. Simulated patient consultation included a 20-min first medical encounter with an actress playing a 38-year-old woman patient. During this consultation, residents had to deliver a breast cancer diagnosis and to discuss treatment (surgery, chemotherapy and radiotherapy). Before the simulated patient consultation, residents had enough time to learn the case description and the aim of the consultation.

### Communication content analysis

The audiotapes of the simulated patient consultations were transcripted. Transcripts were analysed by the LaComm. LaComm is the a French communication content analysis software. This software uses on the one hand a word count strategy based on categories of words such as PROTAN (Hogenraad *et al*, 1995) or Linguistic Inquiry and Word Count (LIWC) (Pennebaker *et al*, 2007) and on the other hand a word combination strategy such as the general inquirer (Stone *et al*, 1966). The aim of this software is to analyse, utterance by utterance, verbal communication used (in medicine in general and in oncology in particular) by identifying utterances types and contents.

Regarding utterances types, communication used during consultations was analysed with the dictionaries included in the LaComm. Dictionaries are composed of words, word stems or expressions. Dictionaries' contents were built on the basis of empirical knowledge derived from actual and simulated patient consultations performed by physicians (Razavi *et al*, 2003; Delvaux *et al*, 2005). The organisation of dictionaries was adapted from the categories of Cancer Research Campaign Workshop Evaluation Manual (CRCWEM) (Booth and Maguire, 1991; Razavi *et al*, 2003; Delvaux *et al*, 2004, 2005) and was redefined and categorised according to the three-function approach of the medical consultation (Cohen-Cole, 1991) by a panel of experts (Table 1). Utterances are thus categorised into three main types: assessment, support and information type.

Regarding utterances' contents, three dictionaries were constructed (medical, emotional and social).

### Breaking bad news process analysis

Transcripts were analysed to test the efficacy of the training programme on the three phases of the BBN process: pre-delivery, delivery and post-delivery phase. To determine these phases, we identified in the transcripts of simulated patient consultations the precise moment when residents broke the cancer diagnosis. Residents were considered to break the cancer diagnosis either when the word cancer was used or when residents confirmed the diagnosis of cancer following a question asked by the simulated patient. So, the pre-delivery phase is the period that spreads from the beginning of the consultation to the beginning of the utterance, in which residents deliver the cancer diagnosis; the delivery phase is the period that consists of the turn of speech, in which residents deliver the cancer diagnosis and the post-delivery phase is the period that spreads from the beginning of the first utterance after diagnosis delivery to the end of the simulated patient consultation.

Efficacy was assessed on time allocated to each of the three phases and on the way residents delivered bad news. The time allocated to these three phases was analysed in seconds. The utterances of the delivery phase were analysed qualitatively. One investigator read all these utterances and assessed whether the diagnosis was delivered precisely. The investigator was masked for time assessment and group allocation.

### Statistical analyses

To be considered for data analysis, physicians had to attend at least 1 h of communication skills training. The data generated from the LaComm are counts of utterance types and contents. The LaComm data and the way the residents delivered bad news (precisely or not) were considered as dependent variables and group-by-time effects were assessed using generalised estimating equation Poisson regression models. The models tested time effects, group allocation effects and also group-by-time effects (training effects) using the training group at baseline and the waiting-list group as the reference group. Changes in the three-phase BBN process were assessed using group-by-time MANOVA. All tests were two-tailed, and *α* was set at 0.05. The analyses were performed with SPSS version 16.0 for PC (SPSS Inc., Chicago, IL, USA).

## Results

### Residents' sociodemographic data

**Figure 1:**
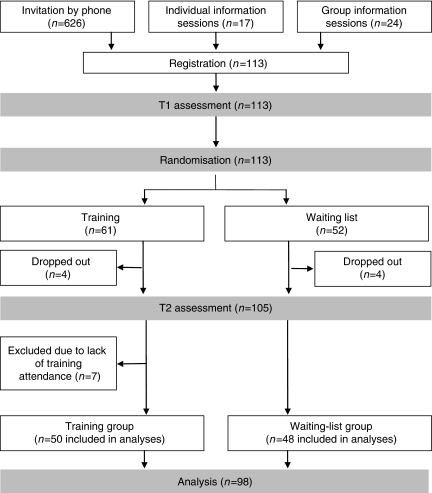
Recruitment procedure, study design, training and assessment procedures. T1, assessments scheduled before the training programme; T2, assessments 8 months after the first assessment.

A total of 113 residents registered to the BIC-CST (Figure 1). Of them, 98 residents completed the simulated patient consultations. Comparison of included and excluded residents showed no statistically significant differences for age, sex and year, and speciality of residency. As to sociodemographic and socioprofessional characteristics, no statistically significant differences were found at baseline between the training-group residents and the waiting-list-group residents except for the type of residency: participants in the waiting-list group are more often residents in oncology (*P*=0.028).

Residents in the training group were a mean of 28 years old (s.d.=3 years), 68% were women, 38% lived alone. Residents were on average in their third year (s.d.=1.3 years) of residency. Among them, 6% were residents in oncology (oncology, haematology and radiotherapy), 32% in gynaecology and 62% in other specialities (surgery, gastroenterology and so on). Five residents had attended a brief communication skills training workshop last year. Residents in the waiting-list group were a mean of 28 years old (s.d.=2.1 years), 60% were women, 25% lived alone. They were on average in their third year (s.d.=1.2 years) of residency. Among them, 25% were residents in oncology, 21% in gynaecology and 54% in other specialities. No resident had attended a brief communication skills training workshop in the past year.

Trained residents underwent on average 25 h of training (s.d.=8.1). They participated in the 8 h of the 10-h stress management skills module (s.d.=2.4) and in the 17 h of the 30-h communication skills module (s.d.=6.8).

### Training effects on residents' utterances

Generalised estimating equation Poisson regression analysis did not show significant group-by-time effects of attendance to BIC-CST on the number of residents' turn of speech but showed significant group-by-time effects on the counts of the types of residents' utterances (Table 2). During the second assessment time compared with baseline, regression analysis showed a significant increase in the rate of open questions (*P*<0.001) and open directive questions (*P*=0.003) for trained residents compared with untrained residents. Analysis also showed a significant increase in the rate of empathy (*P*=0.017). Moreover, analysis showed a significant decrease in the rate of the category ‘other types of information’ (*P*=0.001) and in the total information type (*P*=0.001) for trained residents.

Generalised estimating equation also showed significant group-by-time effects on residents' utterance contents (Table 2). At the second assessment time compared with baseline, regression analysis showed a significant decrease in the count of medical (*P*<0.001), emotional (*P*=0.024) and social (*P*<0.001) words for trained residents compared with untrained residents.

### Training effects on simulated patients' utterances

Generalised estimating equation did not show significant group-by-time effects of attendance to BIC-CST on the number of simulated patients' turn of speech. However, it showed significant group-by-time effects on the number of simulated patients' contents (Table 2). At the second assessment compared with baseline, regression analysis showed a significant increase in the rate of medical (*P*<0.001) and emotional (*P*=0.049) words for the simulated patients when they interacted with trained residents compared with untrained residents.

### Training effects on the three-phase breaking bad news process

Group-by-time effects on the time allocated to each of the three phases of the BBN process were analysed with a MANOVA. Four residents were not included in this analysis. Two in the training group and two in the waiting-list group did not clearly break the cancer diagnosis and the three-phase analysis could not been applied.

**Figure 2:**
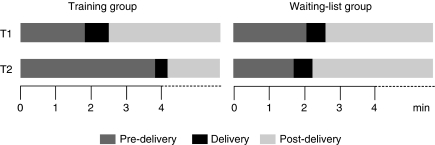
Training effects on the three-phase breaking bad news process.

As shown in Table 3 and in Figure 2, MANOVA showed significant over time and between-group changes in the duration of the pre-delivery phase (*P*<0.001), the delivery phase (*P*=0.009) and the post-delivery phase (*P*<0.001). The pre-delivery phase lasted longer for trained compared with untrained residents. The delivery phase was shorter for trained compared with untrained residents. The post-delivery phase was also shorter for trained compared with untrained residents.

Group-by-time effects on the qualitative content analysis of diagnosis were analysed with GEE. Results showed a significant increase in the transmission of a precise diagnosis among trained residents (RR=3.55; 95% CI=1.21–10.45; *P*=0.021). Indeed, 31 residents in the training group (64.6%) at baseline delivered a precise diagnosis and 41 (85.4%) after training compared with 32 residents in the waiting-list group (69.6%) at baseline and 31 (67.4%) at the second assessment time.

## Discussion

This is the first study assessing in a randomised design the impact of a communication skills training programme on residents' learning of BBN skills. The training programme assessed in this study is a BIC-CST (Bragard *et al*, 2006). Results show that this training programme significantly improves residents' BBN skills.

As to residents' communication skills, it was hypothesised that BIC-CST would lead to an increase in the residents' use of assessment and supportive skills and a decrease in the instances of information transmitted. Results of this study confirm these hypotheses. Trained residents, as expected, used more open and open directive questions, more empathy and transmitted less information after training. Moreover, it should be noted that they also used less emotional, medical and social words. This decrease in residents' number of words can be explained by residents' learning of patient-centred communication skills which requires more function words and less content words. The decrease in the use of content words (medical, emotional and social) may be linked with residents' improvement in communication skills, open and open directive questions requiring less content words. In other words, this residents' learning of patient-centred communication skills leaves more room to patients' expression: results of this study show, as we hypothesised, an increase in the number of emotional and medical words expressed by simulated patients.

As to the BBN process, it was hypothesised that BIC-CST would modify the time allocated to each of the three phases of this process in the following terms: an increase in the duration of the pre-delivery phase and a decrease in the duration of the delivery phase. Results of this study confirm this hypothesis. First, trained residents allocated more time to the pre-delivery phase and less time to the delivery phase. It should be underlined that on average following training the pre-delivery phase increased from 2 to 4 min. Although the effect of this allocation of the time should be tested on patient outcomes, it can be hypothesised that such a lengthening would allow preparation of the patient for BN delivery without unduly increasing this phase and being detrimental to the post-delivery phase. Second, trained residents' bad news delivery was shorter and more precise. Residents should be recalled that BBN is included in nearly all consultations. It should be recalled also that the above three phases are considered in the currently published communication guidelines regarding BBN, but do not always occur in the actual cancer clinical interactions. It should be recalled, moreover, that cancer interactions often involve multiple moments of potential bad news, and therefore the ‘precise moment’ of the bad news interaction may not actually ever occur. Both, the communication skills associated with discussing bad news and the structuring of those skills into three phases may be however used in all clinical interactions. This would allow physicians in all those interactions to inform and support patients in the context of their needs.

The numerous strengths of this study should be underlined. The first strength is the reference to a three-phase BBN model (pre-delivery phase, delivery phase and post-delivery phase) which has been used to design the training programme contents and to measure its efficacy: the development of this three-phase model could be the basis of further studies assessing specifically the physicians' communication in each of these three phases. The second strength is the use of a training programme which has already been assessed for its efficacy in terms of training techniques and duration (Razavi *et al*, 2003). The third strength is the choice of experienced facilitators who have been trained together in the perspective of this study. The fourth strength is the use of a randomised controlled design to assess the efficacy (Merckaert *et al*, 2005). The fifth strength is the use of a standardised simulated BBN task with actresses, which allows a high test–retest validity in a study with repeated measures. The sixth strength is the use of a content analysis software to assess residents' communication skills through the transcript of the simulated BBN task to avoid inter-rator variability.

The study has some limitations. First, even if there is no difference in the use of medical words between the groups at baseline, it should be noticed that there is a higher proportion of oncology residents in the waiting group compared with the training group. It may be hypothesised that oncology residents are more familiar with oncological content of the simulated consultation and may find it harder to reduce medical information. Second, this study reports only the assessment of training based on a computer content analysis. Third, this study has not assessed the transfer of learned BBN skills to clinical practice.

To conclude, this study extends current literature on communication skills in that it shows that communication skills training programmes may improve residents' BBN skills in a simulated task. This study moreover sheds some new light on the BBN process not only in terms of skills learned but also in terms of the process itself that is the structuring of the interactions. This is important to optimise the BBN process by making it more patient centred to allow residents to adapt their consultation to patients' specific needs and preference. It should be recalled that this study was not designed to assess the transfer of learned BBN skills to the workplace. Future studies should thus assess the impact of training in BBN skills on residents' transfer of these skills both to their BBN consultations and to their everyday interactions. These future studies are highly expected before generalising such an intensive – and thus expensive – training programme.

## Figures and Tables

**Table 1:** Description of the utterance types provided by the LaComm (communication content analysis software)

**Utterance types**	**Definitions**	**Examples**
*Assessment*		
Open questions	Assessment of a wide range of issues, concerns or feelings	How are you doing? Tell me.
Open directive questions	More focused assessment of issues, concerns or feelings	Tell me what occurred since the last treatment; What do you feel about it?
Directive questions	Precise assessment of a specific area	Did you begin the treatment? Are you feeling pain?
Leading questions	Assessment of a more precise dimension while suggesting an answer	You do not have pain, don't you?
Checking questions	Checking of information given without seeking further elaboration	Really? Do you understand what I say?
Other types of questions	Assessments not classified by LaComm into one of the previous categories	
		
*Support*		
Acknowledgement	Support by listening to the patient	Mh, Mh; Right; That should not be easy.
Empathy	Support by showing an understanding of the patient's emotional or physical state	I understand that you are distressed; I realize that you have severe pain.
Reassurance	Support by reassuring the patient about a potential threat, discomfort or uncertainty	Don't worry; I will do everything that is possible to help you.
		
*Information*		
Procedural information	Information about orientation and transition of talk in the consultation	I am Doctor x; Please take a seat.
Negotiation	Proposition to the patient taking his/her point of view into account	I suggest we talk about it with your husband.
Other types of information	Affirmative utterances not classified by LaComm into one of the previous categories	

**Table 2:** Training (group-by-time) effects on residents' turn of speech and utterances (number, types and contents) and simulated patients' utterances (number and contents)

	**Training group (*n*=50)**				**Waiting-list group (*n*=48)**				**Generalised estimating equation**		
	**T1**		**T2**		**T1**		**T2**		**Training effects**		
	**Mean**	**s.d.**	**Mean**	**s.d.**	**Mean**	**s.d.**	**Mean**	**s.d.**	**RR**	**95% CI**	***P*-value**
*Number of turn of speech*											
Residents	64	18	70	23	67	20	68	26	1.08	0.94–1.23	0.287
Simulated patients	64	18	70	22	66	20	67	26	1.09	0.95–1.26	0.206
*Residents' utterances*											
*Types*											
*Assessment*											
Open questions^a^	0.2	0.4	0.8	1.1	0.6	1.7	0.5	0.8	5.79	2.24–14.91	<0.001
Open directive questions	3.1	2.0	4.5	3.1	2.8	1.9	2.3	2.1	1.71	1.20–2.45	0.003
Directive questions	6.3	5.2	4.7	2.8	5.5	7.5	4.7	4.1	0.87	0.57–1.34	0.522
Leading questions^a^	0.3	0.7	0.5	0.9	0.2	0.5	0.2	0.4	1.98	0.56–7.02	0.290
Checking questions^a^	2.8	2.6	3.3	4.4	2.1	1.8	2.3	1.8	1.12	0.68–1.87	0.655
Other types of questions	16.5	8.0	21.2	10.3	15.2	7.5	16.3	11.4	1.20	0.90–1.61	0.206
Total	29.1	13.2	35.0	17.7	26.4	12.1	26.2	14.1	1.21	0.97–1.50	0.090
											
*Support*											
Acknowledgement	23.2	14.4	26.6	15.2	23.9	17.2	22.2	14.0	1.24	0.98–1.58	0.077
Empathy^a^	0.2	0.4	0.5	0.9	0.3	0.6	0.2	0.5	4.50	1.31–15.50	0.017
Reassurance^a^	0.7	0.8	0.4	1.0	0.5	0.9	0.7	1.1	0.48	0.18–1.26	0.136
Total	24.0	14.4	27.6	15.2	24.7	17.4	23.0	14.3	1.23	0.98–1.55	0.073
											
*Information*											
Procedural information	7.9	4.0	6.8	2.9	8.5	5.7	8.1	6.7	0.90	0.70–1.15	0.398
Negotiation^a^	1.6	1.8	1.3	1.7	1.6	2.3	1.4	1.9	0.94	0.55–1.62	0.833
Other types of information	53.9	21.5	37.3	22.9	54.7	25.8	55.6	25.5	0.68	0.55–0.85	0.001
Total	63.4	22.5	45.4	24.2	64.8	29.0	64.9	28.5	0.72	0.59–0.87	0.001
											
*Contents*											
Medical words	76.1	24.2	53.8	20.1	78.0	29.1	74.2	23.4	0.74	0.64–0.87	<0.001
Emotional words	10.8	5.7	9.5	6.9	9.2	5.9	10.7	7.8	0.75	0.59–0.96	0.024
Social words	15.2	7.6	9.4	6.3	16.0	7.9	17.7	9.3	0.56	0.45–0.69	<0.001
											
*Simulated patients' utterances*											
*Contents*											
Medical words	24.2	7.1	35.8	10.7	25.9	9.5	25.4	8.0	1.51	1.32–1.73	<0.001
Emotional words	12.0	4.3	12.4	5.0	11.4	4.4	9.9	4.3	1.19	1.01–1.42	0.049
Social words	18.4	5.7	17.6	6.1	17.5	5.4	17.5	5.7	0.96	0.84–1.10	0.578

Abbreviations: CI=confidence interval; RR=relative rate; T1=at baseline; T2=after training for the training group and after 8 months for the waiting-list group.

Estimated relative rates based on a generalised estimating equation Poisson regression model (*n*=98).

a Negative binomial distribution.

**Table 3:** Training (group-by-time) effects on the duration of the three phases of the breaking bad news process: MANOVA (*n*=94)

	**Training group (*n*=48)** a				**Waiting-list group (*n*=46)** a				**MANOVA**			
	**T1**		**T2**		**T1**		**T2**		**Time**		**Group by time**	
	**Mean**	**s.d.**	**Mean**	**s.d.**	**Mean**	**s.d.**	**Mean**	**s.d.**	**F_1.92_**	***P*-value**	**F_1.92_**	***P*-value**
Pre-delivery phase (s)	113	98	235	139	127	142	106	90	12.40	0.001	25.32	<0.001
Delivery phase (s)	42	25	23	18	36	42	33	32	14.09	<0.001	7.09	0.009
Post-delivery phase (s)	1045	106	943	137	1037	138	1061	86	7.36	0.008	19.82	<0.001

Abbreviations: T1=at baseline; T2=after training for the training group and after 8 months for the waiting-list group.

a Two residents were excluded from the analysis because they never expressed the word ‘cancer’ at T2.
